# Impedance Control Method for Tea-Picking Robotic Dexterous Hand Based on WOA-KAN

**DOI:** 10.3390/s25237219

**Published:** 2025-11-26

**Authors:** Xin Wang, Shaowen Li, Junjie Ou

**Affiliations:** Key Laboratory of Agricultural Sensors, Ministry of Agriculture and Rural Affairs, School of Information and Artificial Intelligence, Anhui Agricultural University, Hefei 230036, China; kingxin_wx@163.com (X.W.); oujunjie2025@163.com (J.O.)

**Keywords:** tea-picking robots, dexterous hand, impedance control, neural network, force tracking

## Abstract

Focusing on the mechanical characteristics of robotic dexterous hand tea-picking, this paper takes the harvesting of the premium tea Huangshan Maofeng as an example and proposes an adaptive impedance control method for tea-picking dexterous hands based on the Whale Optimization Algorithm (WOA) and Kolmogorov–Arnold Network (KAN). Firstly, within the impedance control framework, a KAN neural network with cubic B-spline functions as activation functions is introduced. Subsequently, the WOA is applied to optimize the B-splines, enhancing the network´s nonlinear fitting and global optimization capabilities, thereby achieving dynamic mapping and real-time adjustment of impedance parameters to improve the accuracy of tea bud contact force-tracking. Finally, simulation results show that under working conditions such as stiffness mutation and dynamic changes in desired force, the proposed method reduces the overshoot by 14.2% compared to traditional fixed-parameter impedance control, while the steady-state error is reduced by 99.89%. Experiments on tea-picking using a dexterous hand equipped with tactile sensors show that at a 50Hz control frequency, the maximum overshoot is about 6%, further verifying the effectiveness of the proposed control algorithm.

## 1. Introduction

Tea leaves, as an important high-value agricultural product in China, have a distinct harvesting process with unique operational characteristics [1,2,3]. Compared to other crops, tea harvesting has stricter requirements for timeliness and quality control, especially for premium tea products, which demand high precision and standardization in their harvesting process [4,5,6]. Currently, with the continuous advancement of artificial intelligence and robotics technology, significant progress has been made in the research and application of tea-picking robots both domestically and internationally [7,8,9,10,11,12,13,14,15]. Yu et al. [16] proposed a vision-based adaptive oolong tea-picking robot that, by combining visual positioning algorithms and adjustable cutting tools, can adjust the cutting parameters according to changes in the shape of the tea tree and the environment. Wang Lin et al. [17] proposed an intelligent tea-picking robot based on an SCARA robotic arm, which uses a fifth-order polynomial interpolation algorithm for trajectory planning and combines an adaptive robust PD control strategy, allowing the arm to accurately track the target trajectory under external disturbances. For premium tea, although the above-mentioned papers have made significant breakthroughs in visual positioning and trajectory planning for tea-picking robots, they have not considered the requirement for compliance in the end-effector during the harvesting of tender tea shoots.

The dexterous hand, as the core actuator of the tea-picking robot, directly contacts the tea leaves and performs the harvesting task [18,19,20]. Since the dexterous hand picks tender buds and leaves, compliant control is required for the tea-picking dexterous hand [21,22,23,24]. Impedance control, as the core technology for achieving compliant interaction between robots and the environment, has become a research hotspot in the field of robot control since its introduction by Hogan [25]. Traditional impedance control is difficult to meet complex demands due to its poor adaptability to unknown environments, whereas neural networks, with their nonlinear fitting and self-learning capabilities, provide effective solutions for the adaptive adjustment of impedance parameters and improvement in force-tracking accuracy. Currently, some scholars have combined neural networks with impedance control. Dang [26] applied RNN neural networks to impedance control methods, using adaptive PI trajectory compensation and neural network online adjustment of stiffness and damping parameters, improving the force-tracking performance and control compliance of polishing robots under unknown environmental parameters. Huang [27] proposed an adaptive impedance control method based on a broad fuzzy neural network (BFNN), which enhances the modeling ability for unknown environments through broad learning systems, and combines an obstacle Lyapunov function to handle state constraints, achieving compliant control without the need for an environmental model.

The above literature improves traditional impedance control methods through neural networks to achieve force control. However, these methods still have significant drawbacks, such as a complex training process, and like most traditional neural networks, they use gradient descent, which leads to slow convergence, susceptibility to local optima, and difficulty meeting the demand for real-time dynamic adjustment of impedance parameters at high frequencies. In contrast, KAN uses a learnable univariate function as the activation function and deploys it on the edges of the network rather than at the nodes, as in traditional neural networks. The activation functions on each edge of the KAN are formed by B-splines. This structure can smoothly and continuously approximate the nonlinear mapping between force and displacement, thus effectively improving the accuracy of force-tracking in impedance control with respect to environmental changes. At the same time, the WOA is used to adjust the B-spline weight matrix in the neural network online, improving the long learning time and susceptibility to local optimization problems of the KAN neural network. This ensures approximation performance while providing the real–time capability required for compliant tea-picking tasks, enabling effective force control during the picking process.

## 2. Impedance Control Algorithm Based on WOA–KAN Neural Network

### 2.1. Analysis of Impedance Control System

Impedance control establishes a dynamic relationship between contact force and position, enabling the bidirectional transformation between force and motion. The impedance model can be described as follows:

(1) $$M\left({\ddot{x}}_{d}-\ddot{x}\right)+B\left({\dot{x}}_{d}-\dot{x}\right)+\;K\left(x_{d}-x\right)=\;f_{ext}-f_{d}=∆f$$

where $x_{d}$, x denote the desired and actual positions, respectively; M, B, and K represent the desired inertia, damping, and stiffness parameters; $f_{d}$ is the desired finger gripping force vector; $f_{ext}$ is the contact force between the finger and the tea leaves; and ∆f is the force error. 

**Figure 1:**
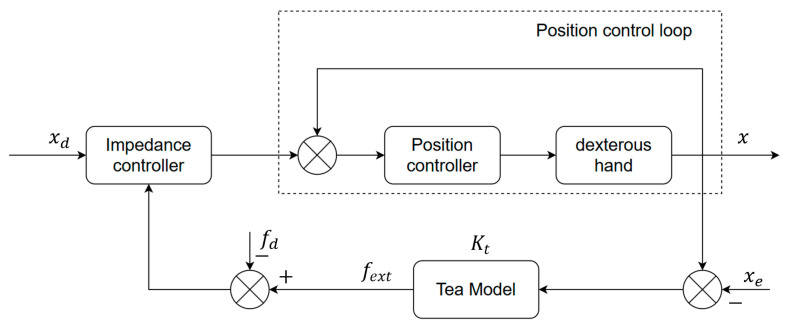
Impedance control block diagram.

The tender shoots of tea leaves (tender buds and leaves) are flexible organ tissues that deform under applied force. They can be equivalently modeled as a first-order admittance model as follows:

(2) $$f_{ext}=K_{t}(x-x_{e})$$

where $x_{e}$ is the initial contact position of the tea leaf, and $K_{t}$ denotes the stiffness of the tea leaf. The block diagram of the impedance control scheme is shown in Figure 1.

Assuming that during the operation of the dexterous hand, the influence of the robot’s inner-loop position control on the system is neglected. To achieve stable gripping of the tea buds by the dexterous hand, it is necessary to meet the force closure condition, which can be derived using the following formula.

(3) $$M\ddot{x}+B\dot{x}+\left(K+K_{t}\right)x=M{\ddot{x}}_{d}+B{\dot{x}}_{d}+Kx_{d}+K_{t}x_{e}+f_{d}$$

(4) $$M\ddot{∆f}+B\dot{∆f}+\left(K+K_{t}\right)∆f\;=K_{t}\left[M\left({\ddot{x}}_{d}-{\ddot{x}}_{e}\right)+B\left({\dot{x}}_{d}-{\dot{x}}_{e}\right)+K\left(x_{d}-x_{e}\right)\right]-\left(M{\ddot{f}}_{d}+B{\dot{f}}_{d}+Kf_{d}\right)$$

When the dexterous finger reaches a steady-state position, both the finger’s velocity and acceleration approach zero.

(5) $$\left(K+K_{t}\right)x_{s}=Kx_{d}+K_{t}x_{e}+f_{d}$$

(6) $$F=K_{t}(x_{s}-x_{e})=\frac{KK_{t}}{K+K_{t}}\;\left[\frac{f_{d}}{K}+{(x}_{d}-x_{e})\right]$$

(7) $$∆f=F-f_{d}=\frac{KK_{t}}{K+K_{t}}\;\left[{(x}_{d}-x_{e})-\frac{f_{d}}{K_{t}}\right]$$

To achieve zero steady-state error, the desired position $x_{d}$ of the dexterous hand must satisfy the following expression:

(8) $$x_{d}=x_{e}+\frac{f_{d}}{K_{t}}$$

When the desired trajectory satisfies (8), the force deviation ∆f approaches zero along the desired trajectory. To directly use (8) to compute the reference trajectory $x_{d}$ and achieve precise force control within a conventional impedance control framework, the tea leaf position $x_{e}$ and stiffness $K_{t}$ are usually required in advance. Although the stiffness of the tea leaf can be estimated experimentally, estimation errors make conventional fixed-parameter impedance control difficult to adapt to variations among different tea leaves, thereby limiting force-tracking accuracy. To address this issue, a WOA–KAN neural network is constructed in this paper to adaptively tune the control parameters. Using tactile sensors, the feedback contact force signals are obtained in real time, and the position deviation between the fingers and the picked tea leaves, together with the contact force deviation, are used as neural network inputs to adjust the desired inertia, damping, and stiffness parameters online. Then, a corrected position is computed according to the impedance control formulation and used as the input to the position control loop, enabling the dexterous hand to dynamically adjust the fingertip position during contact so that it gradually approaches the desired steady-state position $x_{d}\;$, thereby achieving tracking of the desired force without requiring estimation of the tea leaf stiffness. Meanwhile, admissible ranges are imposed on the impedance parameters generated by the WOA–KAN network, and the parametric perturbations of the tea leaf stiffness within the prescribed operating range, together with the uncertainties arising from other unobserved factors, are uniformly modeled as system parametric uncertainties, thereby maintaining a certain passivity margin within this range and ensuring the robustness of the force control performance.

### 2.2. KAN Neural Network

Liu et al. [28], inspired by the Kolmogorov–Arnold representation theorem, proposed the KAN neural network structure in May 2024. The core idea of this theorem is that any multivariate continuous function defined on a bounded region can be expressed through the nesting and addition of a finite number of univariate continuous functions. The Kolmogorov–Arnold theorem can be expressed as follows:

(9) $$f(x)\;=\;f(x_{1},\ldots ,x_{n})\;=\sum_{q=1}^{2n+1}\Phi_{q}\;\left(\;\sum_{p=1}^{n}\phi_{q,p}\;\left(x_{p}\right)\right)$$

In the equation, $\phi_{q,p}\;\left(x_{p}\right)$ is the univariate function corresponding to the q–th node in the hidden layer and the p–th input variable; $\Phi_{q}$ is the operation used to receive and weight-sum the outputs of all hidden layer nodes, thus obtaining the final network output.

From the above equation, it can be seen that any multivariate function can be transformed into the form of a composition and sum of univariate functions. This provides a direct theoretical basis for the network structure of KAN. To eliminate the dependence on the linear weight matrix, KAN uses B-spline functions as the univariate function at each node. The B-spline function is formed by the weighted sum of control points from multiple basis functions, and it is a key parameter that KAN needs to learn during the training process. Its expression is as follows:

(10) $$S\left(x\right)=\sum c_{i}B_{i}(x)$$

In the equation, $c_{i}$ represents the trainable coefficient, and $B_{i}(x)$ is the B-spline basis function defined on the grid. The coefficients $c_{i}$ are optimized during the training process using methods such as the Whale Optimization Algorithm to minimize the loss function.

The KAN uses B-spline functions as activation functions. These B-splines can adjust their shape to adapt to the complex relationships in the data, thereby minimizing approximation errors and enhancing the network´s nonlinear mapping ability and interpretability. The general representation form of KAN is as follows:

(11) $$KAN=\left(\Phi_{L-1}\circ {\;\Phi }_{L-2}\;\circ \;\cdots \;\circ \Phi_{1}\circ \Phi_{0}\right)x$$

Here, $\Phi_{1}$ is the B-spline function matrix corresponding to the first KAN layer; x is the input matrix; ∘ represents the symbol for the inter-layer connection function.

**Figure 2:**
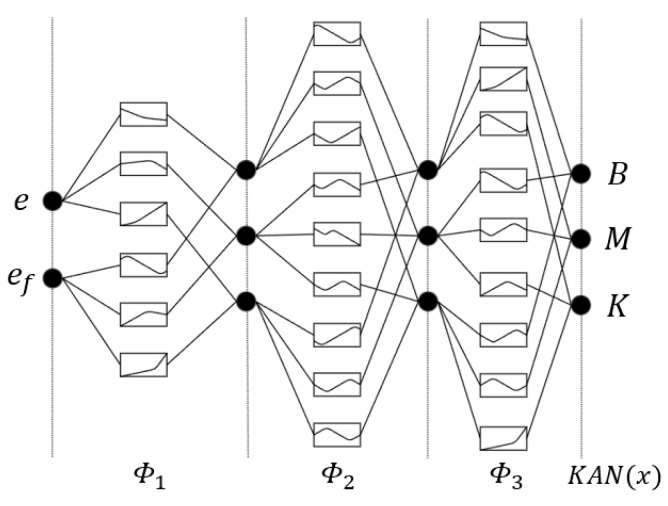
KAN structure.

In the KAN structure, the activation functions are located on the edge connections of the network, while the nodes mainly perform summation operations. The intermediate layer contains 2*n* + 1 variables. To improve numerical approximation accuracy, the KAN can be designed with two or more layers based on practical requirements. In terms of node distribution, a uniform distribution strategy is adopted to ensure that each interval can adequately represent the data variation, thereby flexibly adapting to different application scenarios. Therefore, the KAN neural network is designed as a four-layer network, consisting of an input layer, two hidden layers, and an output layer. The position error $e=x_{d}-x$ and force error $e_{F}=F_{d}-F$ are used as input signals to the neural network. The activation function selected is the cubic B-spline, with 5 B-spline basis functions, and the outputs are the impedance parameters M, B, and K. The KAN structure is shown in Figure 2.

To ensure the stability and accuracy of the KAN neural network in impedance control, several constraint measures have been introduced in the network design. These measures mainly focus on the normalization of input signals, the design of network activation functions, parameter regularization, and physical constraints on the outputs, aiming to improve the training efficiency of the algorithm and avoid overfitting. The detailed explanation is shown in Table 1.

### 2.3. Whale Algorithm Optimizes KAN Neural Network

The Whale Optimization Algorithm (WOA) [29] is a nature-inspired optimization algorithm that simulates the hunting behavior of humpback whales. WOA mimics mechanisms such as encircling, spiral contraction, and random search during the hunting process of individual whales. The position of each whale in the search space represents a set of parameters to be optimized. These parameters are evaluated using a fitness function, and the algorithm iteratively evolves to find the global optimal solution.

**Figure 3:**
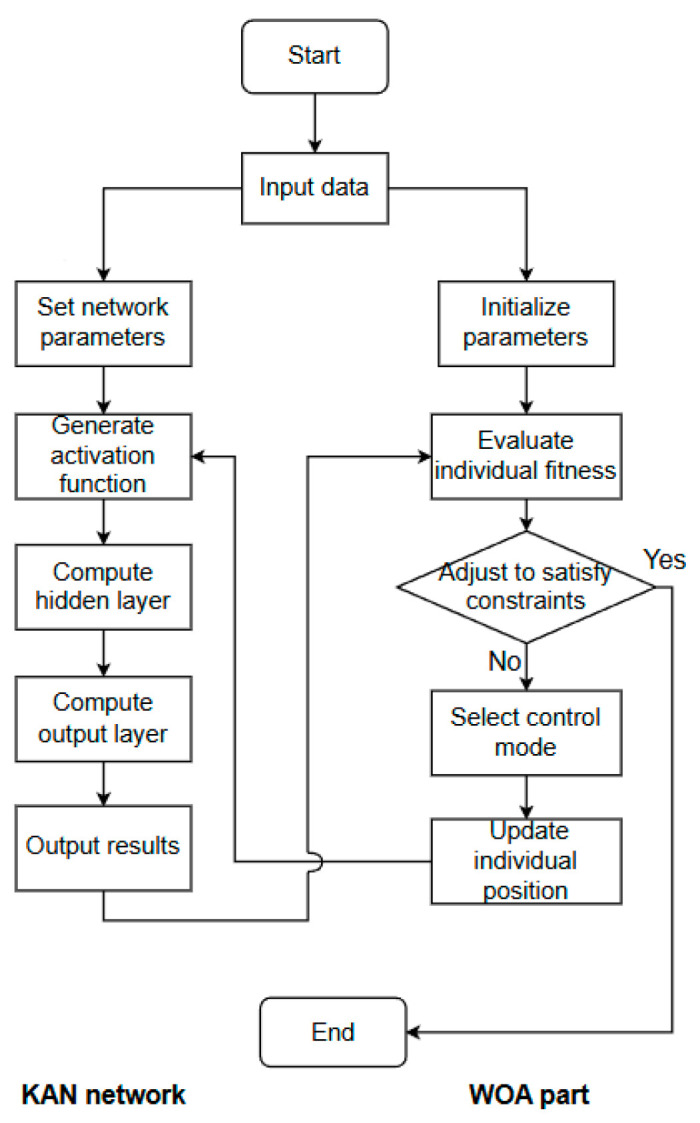
WOA optimizes KAN process.

The WOA–KAN neural network is an algorithm that combines the Whale Optimization Algorithm (WOA) and the KAN neural network. WOA is used to optimize the control points of the spline curve, which serves as the activation function of the network. The control process is shown in Figure 3. First, the population is initialized, control points are generated, and the fitness is calculated. Then, WOA optimizes and gradually updates the control points. Finally, the optimal control points are obtained, allowing the KAN network to output impedance control parameters accurately.

### 2.4. Design of WOA–KAN Impedance Controller

The pseudocode of the WOA–KAN neural network algorithm is shown in Algorithm 1. In flexible operations such as tea harvesting, this method can dynamically adjust the impedance parameters when there is uncertainty in the tea stiffness, thus maintaining a stable force–displacement relationship. The specific step-by-step process is as follows:

Step 1: The input layer has two neurons, corresponding to the position error and force error of the dexterous hand´s end-effector. The input signals to the KAN neural network are the desired trajectory, desired force, end-effector trajectory, and contact force. As shown in Algorithm 1: 4 and 5.

Step 2: The Whale Optimization Algorithm represents the position vector of each whale as a set of B-spline control points, which form a candidate parameter set. The parameter set of the current whale generates a complete B-spline function curve, which serves as the activation function for the network. As shown in Algorithm 1: 6.

Step 3: Each WOA individual corresponds to a specific B-spline curve, which serves as the activation function for the KAN network. The forward propagation of the network will use this B-spline activation function to process the data. The input data is mapped through the KAN´s spline function, then the activation function generated by the B-spline curve is applied, and finally, the KAN output is computed. As shown in Algorithm 1: 7 and 8.

Step 4: The force error is calculated based on the impedance parameters output by the WOA–KAN neural network. The fitness function uses the reciprocal of the mean squared error as the fitness value:

(12) $$fitness=\;\frac{1}{{\left(y_{target}(i)-y(i)\right)}^{2}+1}$$

where $y_{target}(i)$ is the desired force and $y\left(i\right)$ is the predicted force. As shown in Algorithm 1: 9 and 10.

Step 5: The position of the individual is optimized based on the fitness. The individual update formula is the core of the WOA, where the optimal control points are searched through mechanisms such as encircling the prey, bubble-net foraging, and random search. As shown in Algorithm 1: 12.

Step 6: Repeat steps 3 to 5, where WOA iteratively adjusts the B-spline control points through multiple iterations, enabling the generation of better activation functions, thereby optimizing the performance of the KAN in real time. As shown in Algorithm 1: 2 and 3.
**Algorithm 1: WOA-KAN Neural Network Algorithm****Input:** Desired trajectory $x_{d}$, desired force $F_{d}$, current trajectory x, contact force $F_{ext}$ **Output:** Optimized impedance control parameters M, B, K
  1   Initialize neural network parameters and whale algorithm parameters  2   **for** t=1 **to** MaxIter **do**  3     **for each** whale individual p **do**  4       
$e\leftarrow x_{d}-x,\;e_{f}=F_{d}-F_{ext}$           // Position and force errors  5       
$x_{in}\leftarrow (x_{d}-x)/2$  6       
$\Phi_{i}\leftarrow B_{i}\left(x_{in}\right)\;for\;i=1,\ldots ,n$         // B-spline basis computation  7       
$y\leftarrow \sum_{i=1}^{n}a_{i}\Phi_{i}$        // Compute KAN output via weighted B-spline  8       
$y_{1}=M,\;y_{2}=B,\;y_{3}=K$               //  KAN outputs  9       
$y_{target}-y_{pred}\leftarrow M\ddot{x}+B\dot{x}+Kx$            // Compute force error  10      
$fitness=\frac{1}{{\left(y_{target}\left(i\right)-y\left(i\right)\right)}^{2}+1}\;$             // Compute fitness  11    **end for**  12    WOA (X, fitness)      // Update whale positions and the best individual  13  **end for**


To ensure the real-time performance and safety of the impedance controller within the system, the control cycle, computing platform, and latency budget must be planned in a unified manner and aligned with the algorithmic complexity and the network´s computational load. On this basis, the saturation characteristics of the dexterous hand and the joint torque limits should also be considered to prevent control commands from exceeding the hardware capability. The summary is provided in Table 2, where T and N denote the number of WOA iterations and the population size, respectively.

## 3. Experimental Structure and Result Analysis

### 3.1. Tea-Picking Dexterous Hand Configuration

**Figure 4:**
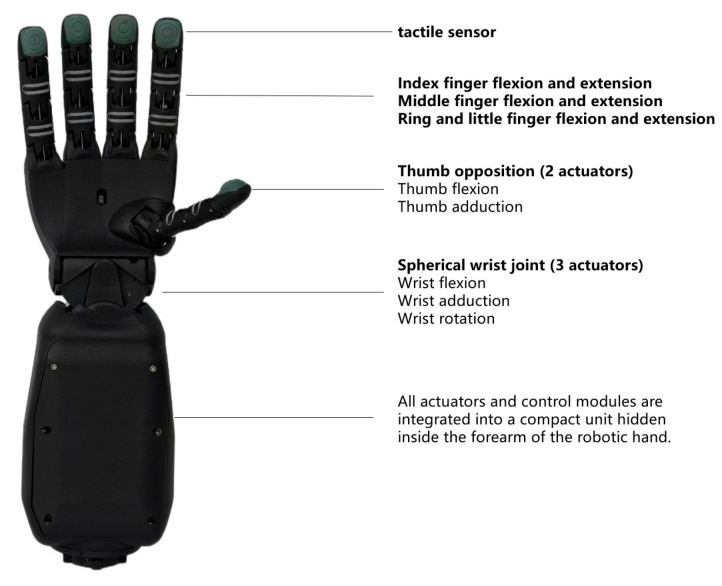
The overall mechanism of dexterous hands for tea-picking.

Premium teas like Huangshan Maofeng have strict harvesting standards, usually only picking one bud and one leaf or one bud and two leaves, while ensuring the tenderness and integrity of the tea leaves. Compared to the limitations of traditional industrial robotic arms with limited degrees of freedom and inadequate force-sensing capabilities, the humanoid dexterous hand, with its human-like structure design and multi-dimensional sensory feedback, is more suited for selecting tea buds and performing compliant operations, meeting the selective and tenderness integrity requirements of premium tea harvesting. Therefore, the humanoid dexterous hand SR–RH8D, developed by Seed Robotics in the UK and manufactured in Lisbon, Portugal, is selected. Its specific features are as follows: The entire system weighs about 620 g, with magnetic connections between the fingers and palm, and it has 19 degrees of freedom (including the opposable thumb and complete spherical wrist joint). It has eight compact actuators with independent control, capable of outputting position, speed, PWM output, and high-resolution current measurement, along with real-time high-frequency feedback data. The fingertip´s FTS–3 3D force tactile sensor can measure forces in any direction from the vertical surface of the finger up to 90 degrees, with a resolution of 1 g/1 mN, a standard measurement range of 0–10 N, and a maximum measurable force of 30 kg. The overall structure of the dexterous hand is shown in Figure 4.

### 3.2. Implementation of Impedance Control for Dexterous Hand

The experimental software system is deployed on the Ubuntu 20.04 operating system and developed in Python3.10 The overall architecture is based on the ‘perception–control’ collaborative design and is divided into two core functional modules. The first is the robot control module, which covers the motion control of the dexterous hand and impedance control force-tracking. This module must first complete serial port selection, baud rate, communication frequency, and other parameter configurations, providing the basic control interface for the dexterous hand to perform harvesting actions and force control adjustments. The second is the tactile information processing module, responsible for real-time reception of the raw data from the fingertip FTS–3 3D force tactile sensor, which is then transmitted to the robot control module to provide perceptual data for the dynamic adjustment of impedance control force-tracking parameters.

**Figure 5:**
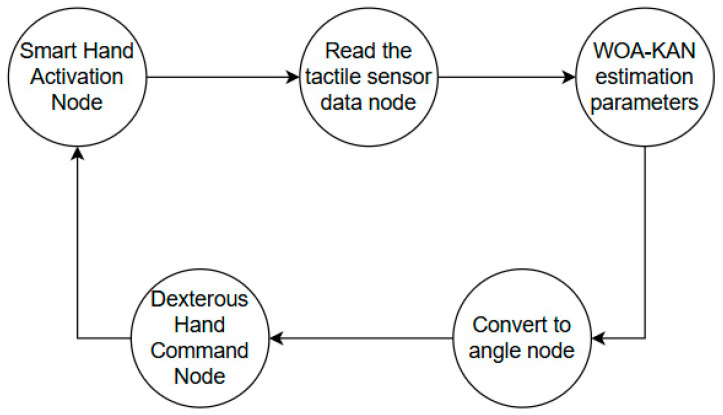
Dexterous hand impedance control process.

The WOA–KAN impedance controller for the dexterous hand is implemented using the Robot Operating System (ROS), with the hardware connection of the dexterous hand and system module initialization handled via launch files. The controller node runs at a frequency of 50Hz, subscribing to the ROS topic for tactile sensor data to obtain real-time contact information. During each control cycle, the system reads tactile data, updates the KAN online via WOA, obtains the optimal impedance control parameters, and calculates the target positions and velocities for each joint of the dexterous hand. After the calculations, the target positions and velocities are published to the SR–RH8D dexterous hand via the corresponding ROS topic, enabling adaptive impedance control based on tactile feedback. The entire control loop operates at a frequency of 50Hz, with an end-to-end delay of approximately 10.475ms from reading tactile data to issuing control commands, ensuring that the system can complete necessary computations and issue control commands within each 20ms control cycle, meeting real-time requirements. The impedance control process for the dexterous hand is shown in Figure 5.

### 3.3. Force-Tracking Simulation Experiment

In the simulation experiments, only the control of the dexterous hand´s end-effector position and force in Cartesian space is considered. To verify the feasibility and effectiveness of the proposed WOA–KAN impedance control system, constant force-tracking and dynamic force-tracking simulations were conducted for the adaptive impedance controller. The main parameter settings of the dexterous hand tea-picking impedance system are as follows: link lengths $m_{1},m_{2},m_{3}\;$= 0.025 m; link masses $m_{1},m_{2},m_{3}\;$= 0.04 kg; desired force $F_{d\;}$= 5 N; desired trajectory $x_{d}=5.25\times 10^{-3}$ m, ${\dot{x}}_{d},{\ddot{x}}_{d}\;$= 0; and tea leaf position $x_{e}=4\times 10^{-3}\;m$. The simulation parameters for the Whale Optimization Algorithm are set as follows: maximum iterations $K_{max}\;$= 50, population size N = 20.

#### 3.3.1. Constant Force-Tracking Simulation Experiment

**Figure 6:**
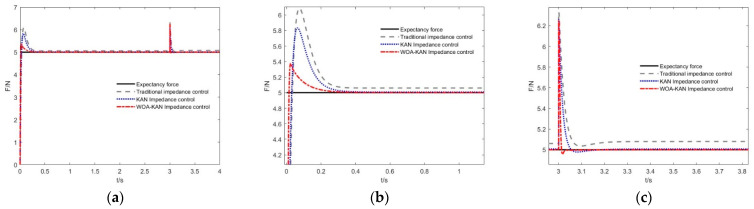
The force-tracking effect of control algorithm under sudden stiffness change. (**a**) Force-tracking effect with abrupt stiffness change. (**b**) Force-tracking effect with a sudden change in stiffness of 4000 N/m. (**c**) Force-tracking effect with a sudden change in stiffness of 6000 N/m.

During tea leaf contact, differences in stiffness among leaves can cause instability in the dexterous hand´s interactions. Under sudden stiffness variations, the robustness of the proposed adaptive impedance control algorithm is evaluated in handling different stiffness conditions. The force-tracking of the fingertip along the x-direction in the inertial coordinate system is shown in Figure 6, where the variation in tea leaf stiffness $K_{t}$ is given by the following expression

(13) $$K_{t}=\left\{\begin{array}{c} 4000\;N/m\;\;\;\;\;t<3\;s\\ 6000\;N/m\;\;\;\;\;t\geq 3\;s \end{array}\right.$$

In a fixed-stiffness tea leaf contact environment, both conventional impedance control and adaptive impedance control can achieve the desired force-tracking performance; however, their force responses still exhibit considerable overshoot. The control method proposed in this paper is capable of achieving the desired force-tracking across tea leaf contact environments with varying stiffness, while maintaining favorable compliance characteristics. The comparative results of the relevant performance parameters are presented in Table 3.

Experimental results demonstrate that, compared with conventional impedance controllers and KAN-based impedance controllers, the proposed WOA–KAN impedance controller achieves the smallest overshoot and steady-state error. Relative to conventional impedance control, the steady-state error is reduced by 99.89% under sudden stiffness variations, while the dynamic overshoot decreases by 14.2%. Compared with the KAN impedance controller, the steady-state error is reduced by 98.75%, and the dynamic overshoot decreases by 9.2%.

#### 3.3.2. Dynamic Force-Tracking Simulation Experiment

During the tea-picking process, force requirements often vary dynamically. To evaluate the adaptability of the control method under dynamic force conditions. In this experiment, the initial desired force was set to $F_{d}\;$= 5 N, and after 3 s, the desired force abruptly changed to $F_{d\;}$= 10N. This experiment was conducted to examine the controller´s response capability under varying desired force conditions and to assess its performance in achieving dynamic force-tracking in complex operational environments.

**Figure 7:**
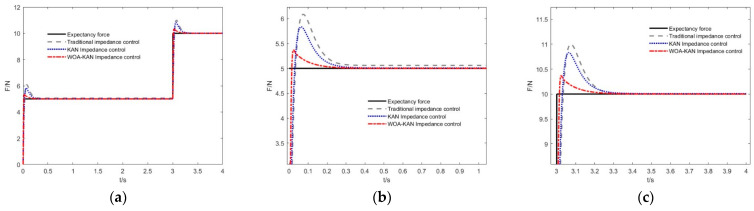
Force-tracking effect of control algorithm under force mutation. (**a**) Force-tracking effect of expected force with abrupt change in fixed-stiffness. (**b**) Force–tracking performance under a desired force of 5 N. (**c**) Force–tracking performance under a desired force of 10 N.

As shown in Figure 7, under varying desired force conditions, both the KAN impedance controller and the WOA–KAN impedance controller exhibit significantly smaller steady-state errors and overshoots compared with the conventional impedance controller. Moreover, the WOA–KAN impedance controller achieves the minimum steady-state error and overshoot under abrupt changes in desired force, resulting in more stable force control.

### 3.4. Dexterous Hand Tea-Picking Experiment

#### 3.4.1. Dexterous Hand Force-Tracking Experiment

**Figure 8:**
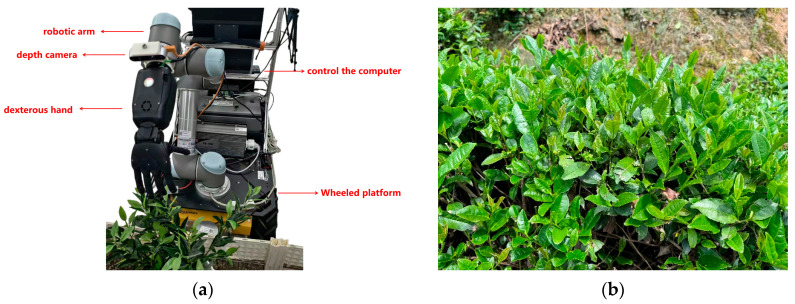
Tea-picking robot and tea garden environment: (**a**) Composition of tea-picking robot system; (**b**) Tea garden environment.

The tea-picking robot is assembled by integrating a Husky wheeled mobile platform, a UR5 collaborative manipulator, a RealSense D435i depth camera, a Seed Hand anthropomorphic dexterous hand, and an upper computer control PC, as shown in Figure 8a. The tea-picking robot has a total of 27 degrees of freedom, including 2 degrees of freedom for the mobile platform, 6 degrees of freedom for the UR5 manipulator, and 19 degrees of freedom for the dexterous hand. Based on this tea-picking robot platform, studies have been conducted in both visual perception and manipulator motion planning, and academic papers have been published in related fields. In terms of visual perception, a visual localization method based on RGB–D information fusion was proposed to address the problem of accurately recognizing and locating picking points for the tea-picking robot in unstructured environments, thereby improving the accuracy of picking point recognition [30]. In terms of manipulator motion planning, an adaptive step motion planning method was proposed for complex tea garden environments, and its improvements in obstacle avoidance capability and trajectory efficiency were verified [31]. On this basis, this paper further investigates the mechanical characteristics of a tea-picking dexterous hand and proposes an adaptive impedance control method for a tea-picking dexterous hand based on a WOA–KAN neural network. Experiments were conducted in a tea garden in She County, Huangshan, Anhui Province, with Huangshan Maofeng as the picking target, as shown in Figure 8b, to verify the effectiveness of the WOA–KAN impedance control algorithm in force-tracking during dexterous hand tea-picking.

**Figure 9:**
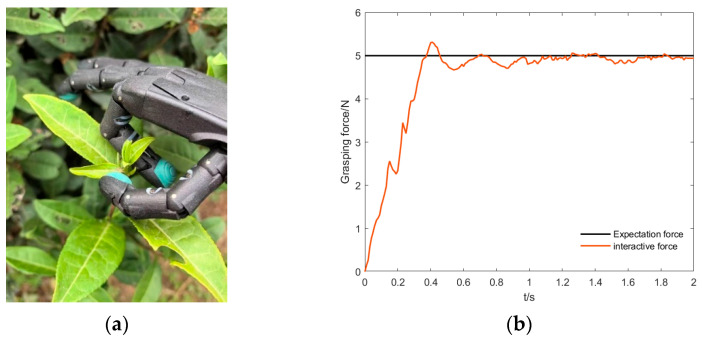
Dexterous hand force-tracking experiment. (**a**) The tea-picking robot´s dexterous hand harvesting tea leaves; (**b**) Tea leaf grasping force impedance control curve.

As shown in Figure 9a, in the dexterous hand tea-picking task, the employed anthropomorphic dexterous hand provides 3–DOF finger motion directions consistent with those of the human finger, enabling imitation of the manual “pinch-and-lift” picking gesture and a grasp that conforms to the tea bud´s morphology. Building on this, the WOA–KAN impedance control algorithm is introduced to enable direction-dependent compliance modulation of the 3-DOF fingers within human-like flexion–extension and lateral planes, while adapting to tea shoot geometric deformation and environmental disturbances during contact and picking. The 3–DOF finger architecture supplies the WOA–KAN impedance controller with control dimensionality and parameter search space, facilitating force–displacement characteristics close to manual picking, thereby improving task success and safety and providing a technical reference for anthropomorphic tea harvesting research.

The force control parameters of the dexterous hand are set as follows: force control sampling interval of 20 ms, grip time of 2 s, and desired gripping force of 5 N.

From the experimental curve of the two-finger tea leaf gripping force, it can be observed that the employed impedance force control algorithm enables the gripping force of the two fingers to track the desired reference force with fast response and low overshoot. As the gripping force deviation decreases, the dexterous hand adaptively adjusts the finger clamping position, thereby ensuring effective force-tracking control and stabilizing the gripping force within the prescribed range. In the experimental process shown in Figure 9b, the maximum force overshoot is 6%, and the system rise time is 0.4 s. Relative to the desired reference force of 5 N, the maximum steady-state error of the two-finger grasping force falls within ±0.3 N.

**Figure 10:**
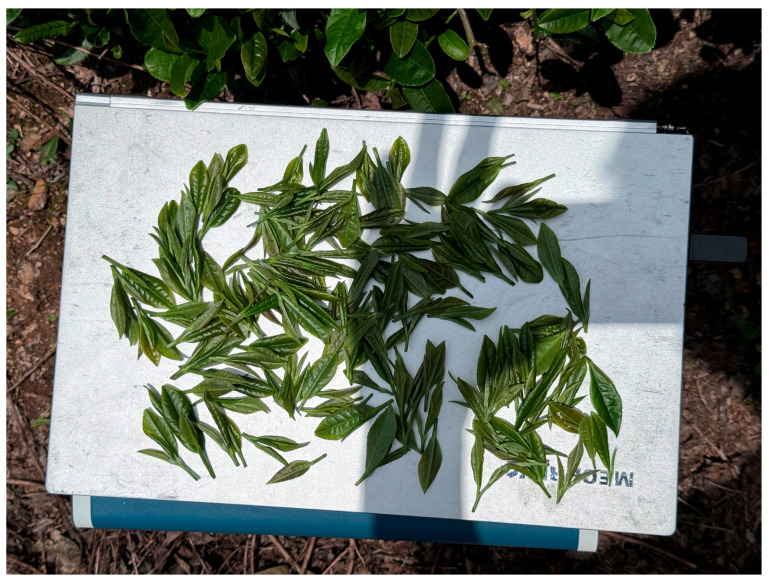
Tea leaf harvest samples.

Multiple picking positions are selected along a single tea row for experiments, where the number of tea buds within the test range is counted, and the system´s tea harvesting rate, usable tea rate, damage rate, and average picking time are evaluated. The usable tea rate is adopted as the evaluation metric, defined as the percentage of usable tea buds relative to the total number of tea buds. The picking experiment results are shown in Figure 10. The detailed experimental results are shown in Table 4.

#### 3.4.2. Environmental Robustness Evaluation

**Figure 11:**
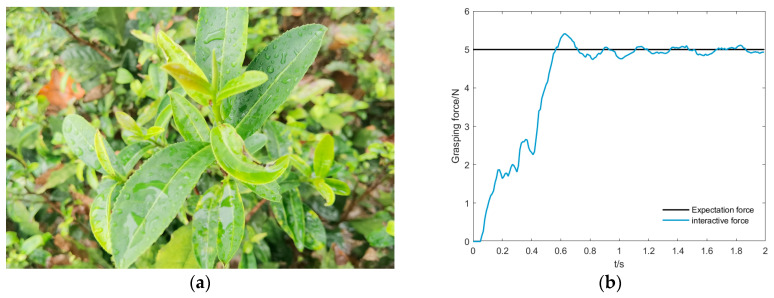
Comparison experiment of tea-picking with wet leaves after rain. (**a**) Post-rain damp tea leaf environment; (**b**) Damp tea leaf grasping force impedance control curve.

The tea-picking experiments conducted in the previous section were carried out under typical picking conditions, such as clear weather and low wind speed. Under special environmental conditions, such as after rain, high humidity, or windy weather, the tactile force sensors may experience additional zero drift due to humidity, and the stiffness and surface friction characteristics of tea buds may change significantly, affecting the calibration accuracy of impedance control parameters and the stability of the picking process. To mitigate the impact of these factors, the system performs zero calibration of the tactile sensors before each operation to ensure the accuracy of force data collection. Additionally, signal filtering and parameter constraints are introduced in the impedance controller to ensure that the inertia, damping, and stiffness parameters generated by WOA–KAN meet stability requirements. For this purpose, the experiment will compare the picking of wet tea leaves after rain to evaluate the force-tracking effectiveness, reliability, and robustness of the WOA–KAN impedance controller under different environmental conditions. The experimental environment and results are shown in Figure 11. The specific experimental results are shown in Table 5.

#### 3.4.3. Discussion on Domain Adaptation

This study employs an impedance controller based on WOA–KAN, using online learning applied to the tea-picking robot’s dexterous hand, and deploying it in the Huangshan Maofeng tea garden scenario. The controller parameters are dynamically updated in real time based on continuous interaction data during the picking process (position error and contact force error). Experimental results show that under most operating conditions, the system achieves good tracking accuracy and contact stability. These error signals inherently reflect the equivalent stiffness and damping characteristics of the bud-stem system, while changes in leaf moisture content and tender stem diameter correspond to changes in equivalent stiffness and damping. When the bud-stem mechanical properties of other tea trees or crop varieties are similar to those of the study subject, feature mapping methods can be used to map the source domain’s characteristics (stiffness and damping) to the feature space of the target domain, enabling the controller to adapt to new input data. The learned online mapping relationship can theoretically be transferred to other varieties without the need for large-scale re-tuning. The WOA–KAN neural network used in the controller can dynamically adjust its network’s activation function by receiving real-time data from the target domain, enabling it to adapt to new tasks and environmental changes. This allows the control system to adjust to the characteristics of the target domain, maintaining efficient system operation. Additionally, constraint measures are introduced for the output of impedance parameters in the controller implementation: reasonable value ranges for the impedance parameters are preset based on the mechanical system characteristics, and boundary constraints are applied to adjust outputs that exceed the range, ensuring the network maintains physical consistency during optimization.

## 4. Conclusions

In response to the force-tracking problem of premium tea under the differing mechanical properties of tea leaves, this paper proposes an adaptive impedance control method based on the WOA–KAN neural network, applied to the tea-picking dexterous hand. The conclusions drawn are as follows:

1. To address the long learning time and overfitting issues of the KAN neural network, the WOA is used to adjust the activation function weight matrix of the neural network online, improving the optimization performance of the KAN neural network.
2. To address the uncertainty caused by unclear tea leaf information during the picking process, the WOA–KAN neural network is combined with impedance control to design the WOA–KAN impedance controller. This controller relies on end-effector force sensors to collect real-time contact force signals between the finger and tea bud, thereby sensing the stiffness differences in different tea leaves online and achieving real-time adaptive adjustment of the system´s inertia, damping, and stiffness parameters, improving compliance and force-tracking performance during the picking process.
3. The simulation results show that, compared to the traditional fixed-parameter impedance controller, the proposed WOA–KAN impedance controller achieves higher end-effector contact force control accuracy, with overshoot reduced by 14.2% and steady-state error reduced by 99.89%.
4. The real-world experiment uses Huangshan Maofeng as the picking target, validating the WOA–KAN impedance control algorithm with a tea-picking robot’s dexterous hand. The results show that at a 50 Hz control frequency, the maximum overshoot during tea-picking is about 6%, with a rise time of 0.4 s and a steady-state error of ±0.3 N, meeting the force control requirements for premium tea-picking.

## Figures and Tables

**Table 1:** Specific constraint conditions description.

Constraints	Description
Input scaling	The position error and force error are normalized, with the position error normalized to the range [−1, 1] and the force error normalized to the range [−5, 5].
Low-pass filtering	A low-pass filter smooths the rate of change in velocity to remove high-frequency noise, and a smoothing saturation function limits the signal, preventing large fluctuations and keeping it continuous and stable.
Regularization constraints	A lightweight L2 regularization constraint is applied to the trainable coefficients with a regularization strength of 1 × 10^−5^ to suppress excessive weight growth, prevent overfitting, and improve the model’s generalization ability.
Smoothing penalty	A smoothing penalty is applied to the output parameters M, B, and K by calculating the sum of the squared increments of the parameters, limiting excessive changes and thereby improving the stability of the model.
Output range control	The impedance parameters M, B, and K output by the network are constrained within specific ranges to ensure that the results comply with physical constraints. The specific output ranges are as follows: M ∈ [0.02, 1], B ∈ [5, 500], and K ∈ [20, 1000].

**Table 2:** Summary of the description of the controller design.

Project	Design Configuration
Control the update frequency	50 Hz
Compute platform	CPU: Intel Core i7-9700 GPU: NVIDIA GeForce GTX 1080 Ti
Latency budget	6 ms
Algorithm running time	8.47 ms
Network computation count	120 times
Complexity per update	O(N × T)
Force saturation	30 N
Torque limits	1.5 Nm
Impedance parameter constraints	M > 0, B > 0, K > 0

**Table 3:** Constant force-tracking effect under sudden stiffness change.

Controller	Maximum Contact Force/N	Steady-State Contact Force/N	Steady-State Force Error/N	Overshoot/%
Traditional impedance controller	6.08	5.06	0.06	21.6%
KAN impedance controller	5.83	5.008	0.008	16.6%
WOA–KAN impedance controller	5.37	5.0001	0.0001	7.4%

**Table 4:** Tea-picking robot dexterous hand harvesting result data.

Number of Experiments	Picked Tea Leaf Count	Usable for Tea Production	Tea Harvesting Rate	Usable Tea Rate	Damage Rate	Average Time
110	94	89	85.45%	80.9%	4.55%	4.54 s

**Table 5:** Comparison of experimental results for picking wet tea leaves after rain.

Condition of Tea Leaves	Rise Time/s	Maximum Contact Force/N	Integral of Absolute Error/N	Overshoot/%
Humid tea leaves	0.55s	5.41	1.75	8.2%
Dried tea leaves	0.4s	5.30	1.05	6%

## Data Availability

As the study data is subject to our laboratory’s confidentiality regulations, disclosure at this stage would violate such regulations.
